# Editorial: the role of diabetes in the pathophysiology and prognosis of ischemic stroke

**DOI:** 10.3389/fendo.2023.1207537

**Published:** 2023-05-04

**Authors:** Juehua Zhu, Yongjun Jiang

**Affiliations:** ^1^ Department of Neurology, The First Affiliated Hospital of Soochow University, Suzhou, China; ^2^ Department of Neurology, The Second Affiliated Hospital of Guangzhou Medical University, Guangzhou, China

**Keywords:** ischemic stroke, diabetes mellitus, insulin resistance, TyG index, reperfusion therapy

Diabetes mellitus is a disease of abnormal carbohydrate metabolism characterized by hyperglycemia. It is associated with a relative or absolute impairment in insulin secretion, along with varying degrees of peripheral resistance to the action of insulin. Diabetes is estimated to affect 537 million adults worldwide, with a global prevalence of 10.5% among adults aged 20 to 79 years (1). Diabetes is one of the most serious and common chronic diseases of our times, causing life threatening disabling complications. Among these complications, stroke is most well-recognized and most common. Stroke is a disease with high prevalence, high disability, high mortality, and high recurrence rate. The lifetime risk of stroke for adult men and women is approximately 25% (2). Worldwide, stroke is the second most common cause of mortality and the second most common cause of disability (3). Diabetes affects 33% of patients with ischemic stroke, and 26% of those with hemorrhagic stroke (4). The Emerging Risk Factors Collaboration meta-analysis of 102 prospective studies with 8.5 million person-years of follow-up demonstrated that diabetes increased ischemic stroke 2.27-fold (5). Diabetes not only affects the onset of stroke, but also is related to the prognosis of stroke outcome. Diabetes double the risk of stroke recurrence, and increases the risk of death or disability post ischemic stroke (6). Diabetic individuals after stroke have a 25% induction in favorable outcome, such as being able to function independently in activities of daily life (7). Furthermore, diabetes has been reported to be associated with increased risk of developing cognitive impairment and dementia after stroke by 2.56-fold (8). Diabetes itself increases the production of reactive oxygen species, promotes the proinflammatory processes. These are the considered mechanisms of accelerated arthrosclerosis and increased risk of thrombus formation which finally lead the onset of ischemic stroke (7, 9). Therefore, in this special issue ‘The role of diabetes in pathophysiology and prognosis of ischemic stroke’ in Frontiers in Endocrinology, we focused on the roles of etiology, pathology, treatment, prognosis of stroke.

Ding et al. explored the association between insulin resistance (IR) and the mechanisms of brain injury after ischemic stroke, which revealed that IR was not a feature of diabetes, but also was essential in the development and progression of ischemic stroke. On one hand, IR added to the risk of embolism by causing endothelial dysfunction and platelet hyperactivation. On the other hand, IR promoted atherosclerosis through inflammatory process, vascular smooth muscle cells proliferation, dyslipidemia, and hypertension. In the clinical practice, there were several well-recognized criteria for evaluating IR, such as homeostasis model assessment of IR (HOMA-IR), oral glucose tolerance tests (OGTT), C-peptide release test, triglyceride glucose (TyG) index, and metabolic score for insulin resistance (METS-IR). And each of them has their own merits and demerits. In recent years, TyG index has been studied as a novel and convenient tool for evaluating atherosclerosis of cardiovascular diseases. In a retrospective observational cohort study of 5593134 persons older than 40 years during a follow-up of 8.2 years, higher TyG was proved to increase the risk of stroke and myocardial infarction by 1.1259- and 1.282-fold (10). In the current issue, Tang et al. demonstrated in a cross-sectional study in southeast China which included 4499 participants aged ≥40 years, that higher TyG index was an independent predictor of the presence of plaque in the carotid artery among the high-stroke-risk population. But there was no correlation between TyG and increased intima-media thickness or carotid artery stenosis. METS-IR is a novel score to screen of evaluate cardiometabolic risk and could be used to screen for early IR as relative to other markers (11). Cai et al. reported that in a retrospective cohort study which included 14032 hospitalized patients, METS-IR was associated with risk of stroke (HR, 1.80; 95CI%, 1.50-2.17) and ischemic stroke (HR, 1.96; 95% CI, 1.60-2.42). Furthermore, circulating microRNAs (miRNAs) have emerged as potential biomarkers in stroke. The downregulation of miRNAs might promote expression of its target genes and their corresponding protein-associated pathways. And miRNA upregulation inhibits expression and the function of target genes and their encoded proteins. Toor et al. specifically emphasized the circulating miRNA profiling of ischemic stroke patients with or without type 2 diabetes. They discovered that Has-miR-361 -3p and -664a-5p were downregulated, whereas miR-423-3p, -140-5p, and -17-3p were upregulated.

Apart from the association of diabetes and incidence of stroke, diabetes is closely correlated to the outcome of stroke. Reperfusion therapy including thrombolysis with intravenous alteplase Tenecteplase, or mechanical thrombectomy for major artery occlusion significantly improves the prognosis of stroke patients (12, 13). Stress hyperglycemia ratio (SHR) defined as defined as the stress fasting glycemia/HbA1c ratio, is a quantitative tool for stress hyperglycemia. Previous studies suggested that high SHR was associated with early neurological deterioration (END) and poor outcome in patients treated with intravenous thrombolysis (14) and increased the risk of symptomatic intracranial hemorrhage and mortality after endovascular thrombectomy (15). In the current issue, Dai et al. demonstrated that SHR increased the incidence of END (OR, 5.77; 95%CI, 1.878-17.742) and decreased the likelihood of favorable outcome (OR, 1.96, 95%CI, 0.077-0.502) in stroke patients with endovascular thrombectomy. Gu et al. from the PostErior circulation iSchemIc Stroke (PERSIST) registry revealed that, for patients with vertebral artery occlusion receiving endovascular treatment, not only SHR, but also admission hyperglycemia, fasting blood glucose (FBG) are predictive risk factor for symptomatic intracranial hemorrhage, poor functional outcomes at 90 days and at 1 year. The effect of these perioperative glycemic indicators was significant in both the general population and the non-diabetic subgroup, but not in the diabetic subgroup. In 955 patients from Nanjing Stroke Registry Program, Wang et al. suggested that elevated TyG index was associated with stroke recurrence within 1 year follow-up (HR, 2.077; 95% CI, 1.158-3.711).

Together, in the Research Topic, we reveal that the status of hyperglycemia, or insulin resistance, increases the risk of atherosclerosis and ischemic stroke, and leads to worse outcome for stroke patients even though reperfusion therapy is applied.

## Author contributions

JZ for manuscript preparation, YJ for revising and final approval.
